# Evolutionary relationships of courtship songs in the parasitic wasp genus, *Cotesia* (Hymenoptera: Braconidae)

**DOI:** 10.1371/journal.pone.0210249

**Published:** 2019-01-04

**Authors:** Justin P. Bredlau, Karen M. Kester

**Affiliations:** 1 Department of Biology, Virginia Commonwealth University, Richmond, Virginia, United States of America; 2 Integrative Life Sciences, Virginia Commonwealth University, Richmond, Virginia, United States of America; Newcastle University, UNITED KINGDOM

## Abstract

Acoustic signals play an important role in premating isolation based on sexual selection within many taxa. Many male parasitic wasps produce characteristic courtship songs used by females in mate selection. In *Cotesia* (Hymenoptera: Braconidae: Microgastrinae), courtship songs are generated by wing fanning with repetitive pulses in stereotypical patterns. Our objectives were to sample the diversity of courtship songs within *Cotesia* and to identify e underlying patterns of differentiation. We compared songs among 12 of ca. 80 *Cotesia* species in North America, including ten species that have not been recorded previously. For *Cotesia congregata*, we compared songs of wasps originating from six different host-foodplant sources, two of which are considered incipient species. Songs of emergent males from wild caterpillar hosts in five different families were recorded, and pattern, frequency, and duration of song elements analyzed. Principal component analysis converted the seven elements characterized into four uncorrelated components used in a hierarchical cluster analysis and grouped species by similarity of song structure. Species songs varied significantly in duration of repeating pulse and buzz elements and/or in fundamental frequency. Cluster analysis resolved similar species groups in agreement with the most recent molecular phylogeny for *Cotesia spp*., indicating the potential for using courtship songs as a predictor of genetic relatedness. Courtship song analysis may aid in identifying closely related cryptic species that overlap spatially, and provide insight into the evolution of this highly diverse and agriculturally important taxon.

## Introduction

Acoustic signals are used by diverse groups of insects for species recognition, fitness displays, and courtship elicitation. Songs used during insect courtship are generally stereotypical within a species and likely play a role in reproductive isolation. Moreover, courtship songs may be a useful identifying character, especially among cryptic or closely related species [1]. For example, songs of *Drosophila* species groups are species-specific and have been studied for evolutionary patterns [2–5]. Furthermore, courtship song analyses have been used in conjunction with genetic, behavioral, and morphological data to reconstruct ancestral songs (e.g., grasshoppers [6]), to revise phylogenies (e.g., a genus of katydids [7]), and to identify cryptic species (e.g., lacewings [8,9] and sand flies [10]). In highly diverse taxa of parasitic wasps, acoustic signals may play a significant role in species differentiation and reproductive isolation.

Male parasitic wasps across multiple families produce wing fanning (also termed “wing vibration”) in response to female olfactory signaling [11–18]. Wing fanning draws air and pheromones over olfactory organs for orientation to the female [19] and likely acts as a display of male fitness. Wing fanning at different amplitudes and velocities generates sound patterns with sound frequency corresponding to wing beat frequency [20]. Wing fanning generates substrate vibrations that are detected by nearby wasps, and substrate type effects mating success [21,22]. Male wing fanning is a necessary precursor for successful mating in many species. For example, in the aphid parasitoid, *Lysiphlebus testaceipes* (Braconidae: Aphidiinae), females mate only after wing fanning and are more likely to mate with males producing higher frequency and higher amplitude wing movement [23].

Wing fanning by parasitic wasps produces patterns of repeating pulses or buzzes, which together comprise a courtship song. For example, five genera of dipteran parasitoids in the subfamily Opiinae (Braconidae) produce songs with short repeating pulses of 40–200 ms at a frequency of 128–190 Hz [24–26]. The aphid parasitoid, *Aphidius ervi* (Braconidae: Aphidiinae), produces repeating pulses lasting ~200 ms at 180 Hz with a ~200 ms pause between pulses [27]. Parasitoids of lepidopteran larvae within the Microgastrinae (Braconidae) produce courtship songs that are formed from a combination of low-amplitude and high-amplitude elements corresponding to changes in frequency [24]. For example, *Glyptapanteles flavicoxis* produces songs that consist of low-amplitude “percussion clicks” from wing vibrations followed by higher amplitude wingbeats increasing in frequency before transitioning back [28]. Considering estimates of 17,000–48,000+ species in Microgastrinae [29], the diversity and evolution of song patterns is almost entirely unknown. Investigating multiple courtship songs within one diverse genus that includes closely related cryptic species, phylogenetic data, and well-characterized model species would provide insight into the general patterns, diversity, and evolution of wasp courtship songs.

The large Microgastrinae genus *Cotesia* contains several species that have served as globally-important biocontrol agents of agricultural pests and as model systems for understanding host-parasitoid and tri-trophic interactions. For example, *Cotesia sesamiae* is a major biocontrol agent of maize stemborers in Africa, and long-term studies have revealed patterns driving host-associated specialization and co-evolution of virulent bracovirus genes [30]. *Cotesia rubecula* and *C*. *glomerata*, both successfully introduced to parts of North America to control the imported cabbageworm, *Pieris rapae* (e.g. [31]), have served as models for parasitoid behavior [13,32]. *Cotesia congregata* is a model system for studying tri-trophic interactions [33,34], insect learning [35–37], insect immunology [38,39], and the genomics of symbiotic bracoviruses [40,41]. Courtship behavior of some *Cotesia* species has been studied to improve mass rearing in biological control programs (e.g. [21,42]). However, most species remain taxonomically undescribed and limited information is available beyond descriptions, host usage, and ranges for the majority of described species.

Courtship songs have been characterized in detail for four species of *Cotesia* [20,24,43]; however, no comparisons have been made among distantly related species, and the songs of most species clusters remain unknown. The most recent phylogeny of *Cotesia* based on four genes contains nineteen *Cotesia* species, several of which are common and well-studied [44]. This phylogeny provides a basic evolutionary framework for comparing courtship songs in *Cotesia*. Moreover, *C*. *congregata*, which is reported to parasitize at least fourteen Sphingidae species that feed on different plant families [45,46], offers an opportunity to compare courtship songs among multiple host-foodplant complex sources. Wasps from two of these host-foodplant complex sources, *Manduca sexta* on tobacco (“MsT”) and *Ceratomia catalpae* on catalpa (“CcC”), have diverged genetically and are likely incipient species [47]. These wasps display a lower male response rate to the female pheromones of the reciprocal source, slight differences in duration and frequency of some song elements, and typically produce sterile hybrid females resulting from CcC♂xMsT♀ crosses [15]. In this study we describe the courtship songs of ten additional species of *Cotesia*, and use clustering to explore the relationships and patterns among songs. Further, we identify song differences among select host-associated populations and incipient species of *C*. *congregata*.

## Materials and methods

### Parasitic wasp collection

*Cotesia spp*. were primarily collected from wild caterpillar hosts at multiple sites in the United States; some *C*. nr. *phobetri* came from an ongoing laboratory colony (Table 1). Caterpillars known to be hosts of *Cotesia* were targeted for collection, particularly *Cotesia* that have published gene sequences. When possible, wasps from different sites were collected for wider population sampling. In most cases, each wasp species came from a single host species. In contrast, *C*. *congregata* were collected from six different sphingid host species feeding on different plant families (Table 1). All permissions were obtained as necessary for field collections on both public and private land from property owners and managers. None of the species involved are listed as endangered or protected.

**Table 1:** *Cotesia* species collected, host-foodplant complex (for *C*. *congregata*), lepidopteran host names, collection locations (county/city, state; lat, long, datum: WGS84), and number of wasp broods collected at each site. *Cotesia congregata* is divided into six host-foodplant complexes (H-FPC) abbreviated by host species and plant name (tobacco, catalpa, holly/pawpaw, Virginia creeper, and privet, respectively).

Wasp species	H-FPC	Host species	Location	Coordinates		*N*
*C*. *congregata* (Say)	MsT	*Manduca sexta* (Linnaeus)	Nottoway Co., VA	37.095	-77.963	8
			West Lafayette, IN	40.287	-86.883	4
	CcC	*Ceratomia catalpae* (Boisduval)	Cumberland Co., VA	37.7127	-78.1639	20
	DhH	*Dolba hyloeus* (Drury)	Chesterfield Co., VA	37.453	-77.581	1
			Hanover Co., VA	37.731	-77.713	1
	DmV	*Darapsa myron* (Cramer)	Gloucester Co., VA	37.304	-76.498	1
			Gloucester Co., VA	37.257	-76.453	2
			Richmond, VA	37.530	-77.450	1
	EpV	*Eumorpha pandorus* (Hübner)	Gloucester Co., VA	37.257	-76.453	2
			Richmond, VA	37.530	-77.450	1
			Henrico Co., VA	37.586	-77.543	1
			Richmond, VA	37.5498	-77.4574	1
	SkP	*Sphinx kalmiae* Smith	Charles City Co., VA	37.331	-77.210	1
*C*. *diacrisiae* (Gahan)		*Estigmene acrea* (Drury)	Goochland Co., VA	37.646	-77.984	3
			Richmond, VA	37.529	-77.453	1
*C*. *empretiae* (Viereck)		*Acharia stimulea* (Clemens)	Richmond, VA	37.519	-77.470	3
			Richmond, VA	37.520	-77.466	1
			Richmond, VA	37.528	-77.455	2
*C*. *euchaetis* (Ashmead)		*Euchaetes egle* (Drury)	Richmond, VA	37.539	-77.451	6
			Richmond, VA	37.524	-77.469	1
*C*. *flaviconchae* (Riley)		*Colias eurytheme* Boisduval	West Lafayette, IN	40.503	-87.013	3
*C*. *glomerata* (Linnaeus)		*Pieris rapae* (Linnaeus)	Richmond, VA	37.6072	-77.5078	1
			Henrico Co., VA	37.595	-77.552	1
			Wellington, CO	40.653	-104.99	1
*C*. nr. *phobetri*		*Grammia incorrupta* (Edwards)	Pima Co., AZ*	32.31	-110.60	2
		*Grammia virgo* (Linnaeus) ^†^	Gloucester Co., VA	37.304	-76.497	3
*C*. *orobenae* (Forbes)		*Evergestis rimosalis* (Guenée)	Goochland Co., VA	37.646	-77.210	23
			Richmond, VA	37.5360	-77.4127	1
			Richmond, VA	37.519	-77.470	2
*C*. *phobetri* (Rohwer)		*Halysidota harrisii* (Walsh)	Richmond, VA	37.520	-77.466	2
			Richmond, VA	37.528	-77.455	4
			Richmond, VA	37.518	-77.474	1
			Richmond, VA	37.549	-77.516	1
*C*. *rubecula* (Marshall)		*Pieris rapae* (Linnaeus)	West Hampton, MA	42.3	-72.8	1^‡^
			St. Paul, MN	44.9	-93.1	1^‡^
*C*. *teleae* (Muesebeck)		*Antheraea polyphemus* (Cramer)	Richmond, VA	37.528	-77.454	1

*Original collection site; wasps for this study were supplied by an ongoing laboratory colony (M. Singer Lab).

^†^ Cocoons at this site were separate from any host. This is the presumed host species found in an adjacent field.

^‡^ Four additional wasps were F_1_ hybrids between these two sites. Collection coordinates are approximate.

Caterpillars, usually collected before parasitization status was known, were reared on their host plant in plastic containers under ambient laboratory conditions until parasitoid egression or pupation. Individual unattached *Cotesia* cocoons were placed in clear gel capsules (size 00) 2–4 days after egression. *Cotesia* species forming a connected cocoon mass were chilled upon adult emergence and placed in individual capsules or vials. Adults were sexed under a dissecting microscope. Wasp songs were recorded within 24 hours of emergence. Voucher samples of each species were both point pinned and stored in 95% EtOH at -20°C. The song of one species included in analysis (*Cotesia marginiventris*) was obtained from a USDA-ARS sound library (https://www.ars.usda.gov/ARSUserFiles/3559/soundlibrary.html) and originally described by Sivinski & Webb [24].

### Audio recordings

Males in capsules were randomly selected from each brood for recording. Individuals were placed in an open paper arena with a drop of honey as a food source to encourage them to stay in the arena. Courtship songs were induced by exposing individual males to an immobilized female of the same species. Songs were recorded using a miniature omnidirectional microphone (model 4060, DPA, Longmont, CO; 20–20,000 Hz) positioned 5–7 mm above the male and a high resolution digital audio recorder (model 702, Sound Devices, Reedsburg, WI; 48 kHz sampling rate, 24 bit resolution) in a sound isolation booth (Industrial Acoustics, Bronx, NY) at 23 ± 1.5°C and 40–55% RH. Generally, one recording per brood was analyzed; however, recordings of different individuals were analyzed for species with fewer than four collected broods. Additional individuals of *C*. *congregata* were recorded to test for relatively small differences among host-foodplant sources. Additional *C*. nr. *phobetri* were recorded from each brood because they could not initially be identified to a known species. Duration of song elements and fundamental frequency were quantified using Raven Pro v1.3 [48]. Waveforms were high-pass filtered at 100 Hz to reduce background noise.

**Fig 1:**
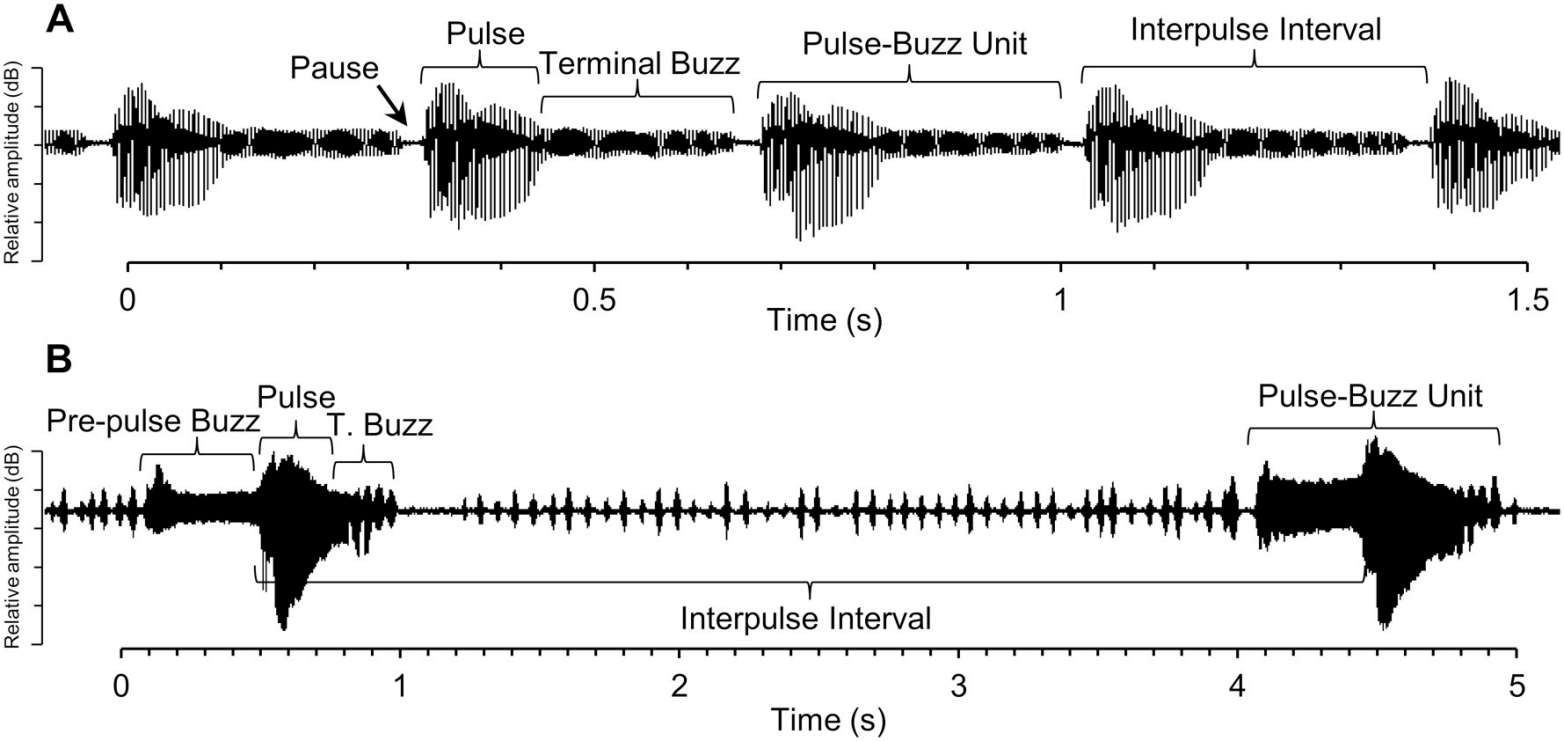
Courtship songs of *Cotesia* species divided into acoustic elements based on relative amplitude and position for use in analysis and comparisons. Represented are typical waveform segments of (A) *C*. *congregata* and (B) *C*. *flavichonchae*.

Songs were divided into multiple elements based on acoustic characteristics shared across species (Fig 1). “Pauses” occur between other song elements when wings are held motionless above the body and do not generate sound. “Pulses” are high amplitude elements comprising the greatest range of wing movement, referred to as “boings” in *C*. *congregata* [20]. “Terminal buzzes” immediately follow pulses and consist of continuous lower-amplitude sounds. “Pre-pulse buzzes” are steady lower amplitude sounds that precede a pulse with no pause in between. For “pulse-buzz units” we measured the duration of the pulse and buzz together. “Interpulse interval” is the time from the start of one high-amplitude pulse to the start of the next. Pulses, buzzes, and pauses make up the pulse-buzz units and interpulse interval, which were included because they make discrete units that may be important in species recognition. Song amplitude was not compared because distance from the microphone varied slightly among some species. Quantification of song elements started with the second complete pulse-buzz cycle and continued for six complete pulse-buzz cycles. Spectrograms of the entire song were produced using a short-time Fourier transform (Hann window, size = 2,000 samples, 50% overlap). Frequency spectra for song sections were calculated using fast Fourier transforms (Hann window, size = 1,000 samples, 50% overlap). Frequency of the first harmonic (fundamental frequency) was used in all comparisons.

### Comparisons and analysis

Several statistical procedures were used to determine differences in courtship songs, consolidate elements, and group songs by structure. The considerable differences among some songs present a challenge for direct comparisons using standard statistical tests. For example, some elements were drastically reduced or absent in some species. For statistical tests, the repeating song elements were averaged so that each individual wasp was treated as an *N* of 1. Where appropriate in similar species, duration and fundamental frequency were compared using analysis of variance (ANOVA) followed by Tukey’s post-hoc test. Song element durations were log transformed to meet the assumptions of ANOVA. Pulse and terminal buzz frequency were compared between adjacent elements of each song for each species and all species together using linear regression.

Principal components analysis (PCA) for each individual wasp was used to condense dimensionality of the data into principal components (PCs) based on communitive explained variance. Hierarchical cluster analysis (Ward method) of mean principal components of each species was used to group wasps. The resulting dendrogram was compared to the most recent molecular phylogeny of *Cotesia* [44] to determine if the same groups were resolved (see Table 2 for genes used and GenBank ascension numbers of species included in this study).

**Table 2:** Available genes and GenBank ascension numbers of *Cotesia* species used in this study. Not all species have been sequenced using all four genes, and therefore are not included in the phylogeny.

Species	NADH1	mt16S rDNA	n28S rDNA	LW rhodopsin
*C*. *congregata*	AF069198	U68157	AJ535936	AJ535980
*C*. *diacrisiae*	AJ535959	AJ535917	--	--
*C*. *empretiae*	AJ535961	AJ535919	AJ535939	AJ535983
*C*. *euchaetis*	AJ535962	AJ535920	AJ535940	AJ535984
*C*. *flaviconchae*	AJ535963	AJ535921	AJ535941	AJ535985
*C*. *glomerata*	AF110830	U68158	AJ535944	AJ535988
*C*. *marginiventris*	AJ535967	AJ535926	AF102730	AJ535991
*C*. *nr phobetri*	--	--	--	--
*C*. *orobenae*	AJ535970	U68158	--	--
*C*. *phobetri*	--	--	--	--
*C*. *rubecula*	AF110831	U06959	AJ535949	AJ535994
*C*. *teleae*	--	--	--	--

Subsequent PCAs were performed on six different host-foodplant complex sources of *C*. *congregata*, on the MsT and CcC incipient species, and the two geographically isolated sources of *C*. nr. *phobetri*. Sources that displayed separation in the PCAs were followed by a Welch’s unequal variance t-test to compare duration and frequency of song elements. The feasibility of matching songs back to their species or population based on song elements and PCs was tested using linear discriminant analysis. All statistical and multivariate analyses were performed with JMP v11 (SAS Institute, Cary, NC).

## Results

### Description of wasp songs

Eleven species of *Cotesia* were collected at different sites in the United States (Table 1); one additional species was from a published recording (*C*. *marginiventris*). Some target host species collected did not yield any *Cotesia*. Multiple broods were collected of each species except for the uncommon *C*. *teleae*, which came from a single *Antheraea polyphemus* larva that produced only one living male concurrently with females. *Cotesia congregata* were collected from six different sphingid host species. *Cotesia* nr. *phobetri*, (currently undescribed) were supplied from a laboratory colony originating from host *Grammia incorrupta* (formerly *G*. *geneura*) that feed on forbs in grassland habitat and found in Redington Pass, Pima County, AZ [49]. Initially unknown specimens found as cocoon clusters independent of hosts in a recently mowed horse pasture in Gloucester County, VA, were identified to be either *C*. nr. *phobetri* or a closely related sister species based on morphology, cocoon structure, similarity of habitat, and song structure (Discussion, 8^th^ paragraph). Unparasitized caterpillars of *Grammia virgo* were found in an adjacent field, which suggest this species is the probable host. The other *Cotesia* species in our study were each collected from a single host species.

**Fig 2:**
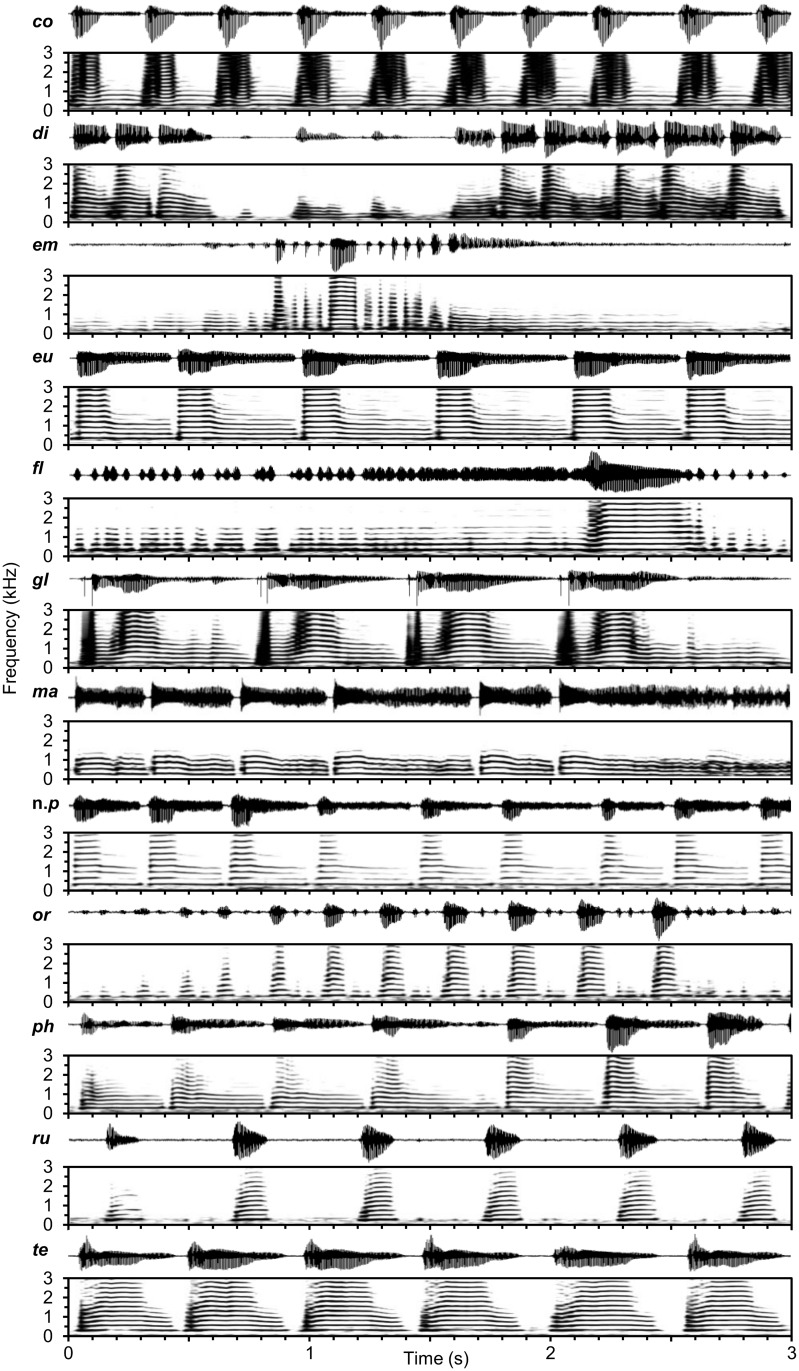
Waveforms and spectrograms of typical courtship songs of twelve species of *Cotesia*. Species are labeled by the first two letters of the species name.

All twelve species of *Cotesia* generated songs by wing fanning that consisted of repeating high-amplitude pulses and, in most species, lower amplitude buzzes (Fig 2). Pulses were accompanied by abdominal movements. All males continued to produce songs until copulation or the female moved away; therefore, number of pulses was not used as a factor. Songs varied in duration of pulse, buzz, and pause elements (Table 3). Some songs were different enough from the others as to not require statistical comparisons of individual elements, e.g., *C*. *flavicochae* produces a song with unique elements. Moreover, songs with considerably longer element durations had greater variance than songs with relatively short element durations (e.g., compare pause duration between *C*. *teleae* and *C*. *congregata* in Table 3), resulting in violation of the assumptions for ANOVA. Although an ANOVA could not be performed with all 12 species, an ANOVA was performed with four species that had song elements of similar durations and frequencies. Among all species, fundamental frequency ranged from 176 Hz in *C*. *phobetri* to 328 Hz in *C*. *euchaetis* (Table 4). All songs produced detectable harmonics up to 4–5 kHz for pulses and 1–2 kHz for buzzes (Fig 2). Analysis of individual elements was useful for comparing similar or sister species but of limited use for comparisons across all species. Courtship songs were divided into groups using a combination of element duration and frequency, and general patterns rather than focusing on individual song elements.

**Table 3:** Duration (ms; mean ± SE) of male courtship song elements among species of *Cotesia*. *Cotesia congregata* is divided by host-foodplant complex. *N* = number of individual wasps analyzed.

Wasp species	Pause	Pulse	Terminal buzz	Pulse-buzz	Interpulse interval	*N*
*C*. *congregata*—MsT	23.4 ± 0.3	133 ± 2	206 ± 3	339 ± 3	362 ± 3	12
*C*. *congregata*—CcC	23.0 ± 1.1	147 ± 1	209 ± 2	356 ± 3	378 ± 3	20
*C*. *congregata*—DhH	25.2 ± 0.6	128 ± 4	216 ± 9	344 ± 11	369 ± 11	3
*C*. *congregata*—DmV	20.8 ± 0.3	126 ± 2	202 ± 4	328 ± 4	349 ± 4	19
*C*. *congregata*—EpV	23.3 ± 0.3	131 ± 1	229 ± 4	361 ± 4	385 ± 4	31
*C*. *congregata*—SkP	20.0 ± 0.7	145 ± 3	205 ± 4	349 ± 5	368 ± 5	1
*C*. *diacrisiae*	26.9 ± 0.8	92 ± 13	109 ± 4	168 ± 5	200 ± 5	4
*C*. *empretiae*	30.3 ± 1.5	54 ± 5	20 ± 8	66 ± 8	100 ± 7	6
*C*. *euchaetis*	37.8 ± 5.8	128 ± 6	281 ± 11	409 ± 14	454 ± 13	5
*C*. *flaviconchae*	0.0 ± 0.0	212 ± 27	240 ± 24	916 ± 38	6056 ± 483	4
*C*. *glomerata*	2.6 ± 1.0	176 ± 12	520 ± 17	696 ± 19	707 ± 19	4
*C*. *marginiventris*	31.2 ± 2.1	98 ± 15	276 ± 45	375 ± 52	405 ± 53	1
*C*. nr. *phobetri*	34.3 ± 0.5	118 ± 3	205 ± 6	323 ± 8	358 ± 8	16
*C*. *orobenae*	38.9 ± 2.3	65 ± 3	30 ± 3	95 ± 2	265 ± 7	6
*C*. *phobetri*	34.5 ± 1.2	109 ± 3	315 ± 10	424 ± 11	454 ± 9	7
*C*. *rubecula*	197.2 ± 19.3	102 ± 2	96 ± 16	198 ± 17	484 ± 8	6
*C*. *teleae*	155.7 ± 22.3	74 ± 2	293 ± 10	368 ± 11	517 ± 15	1

**Table 4:** Fundamental frequency (Hz; mean ± SE) of male courtship song elements among species of *Cotesia*. *Cotesia congregata* is divided by host-foodplant complex. *N* = number of individual wasps analyzed.

Wasp species	Pulse	Terminal buzz	Pulse-Buzz	*N*
*C*. *congregata*—MsT	220.9 ± 1.2	230.6 ± 0.9	223.5 ± 0.9	12
*C*. *congregata*—CcC	222.2 ± 1.3	239.3 ± 1.2	228.7 ± 1.3	20
*C*. *congregata*—DhH	216.9 ± 3.6	231.5 ± 1.3	220.6 ± 2.9	3
*C*. *congregata*—DmV	203.8 ± 1.8	220.6 ± 2.1	206.1 ± 1.8	19
*C*. *congregata*—EpV	212.9 ± 1.6	229.1 ± 1.0	218.6 ± 1.4	31
*C*. *congregata*—SkP	220.9 ± 1.7	230.9 ± 0.6	223.8 ± 1.4	1
*C*. *diacrisiae*	258.8 ± 1.3	241.3 ± 1.9	250.1 ± 1.8	4
*C*. *empretiae*	254.3 ± 3.1	220.7 ± 4.7	252.8 ± 3.3	6
*C*. *euchaetis*	302.2 ± 2.4	271.4 ± 3.1	282.0 ± 5.1	5
*C*. *flaviconchae*	265.9 ± 3.1	294.7 ± 3.5	285.7 ± 2.3	4
*C*. *glomerata*	214.2 ± 1.4	262.9 ± 2.9	224.6 ± 2.8	4
*C*. *marginiventris*	293.3 ± 2.8	247.5 ± 3.5	248.6 ± 4.9	1
*C*. nr. *phobetri*	303.7 ± 1.4	285.3 ± 1.2	294.0 ± 1.5	16
*C*. *orobenae*	277.4 ± 2.0	282.9 ± 2.5	280.1 ± 2.2	6
*C*. *phobetri*	260.5 ± 3.0	228.0 ± 4.6	230.4 ± 4.8	7
*C*. *rubecula*	235.2 ± 2.8	235.2 ± 4.3	235.7 ± 3.0	6
*C*. *teleae*	227.0 ± 1.6	243.9 ± 2.2	231.7 ± 1.8	1

**Fig 3:**
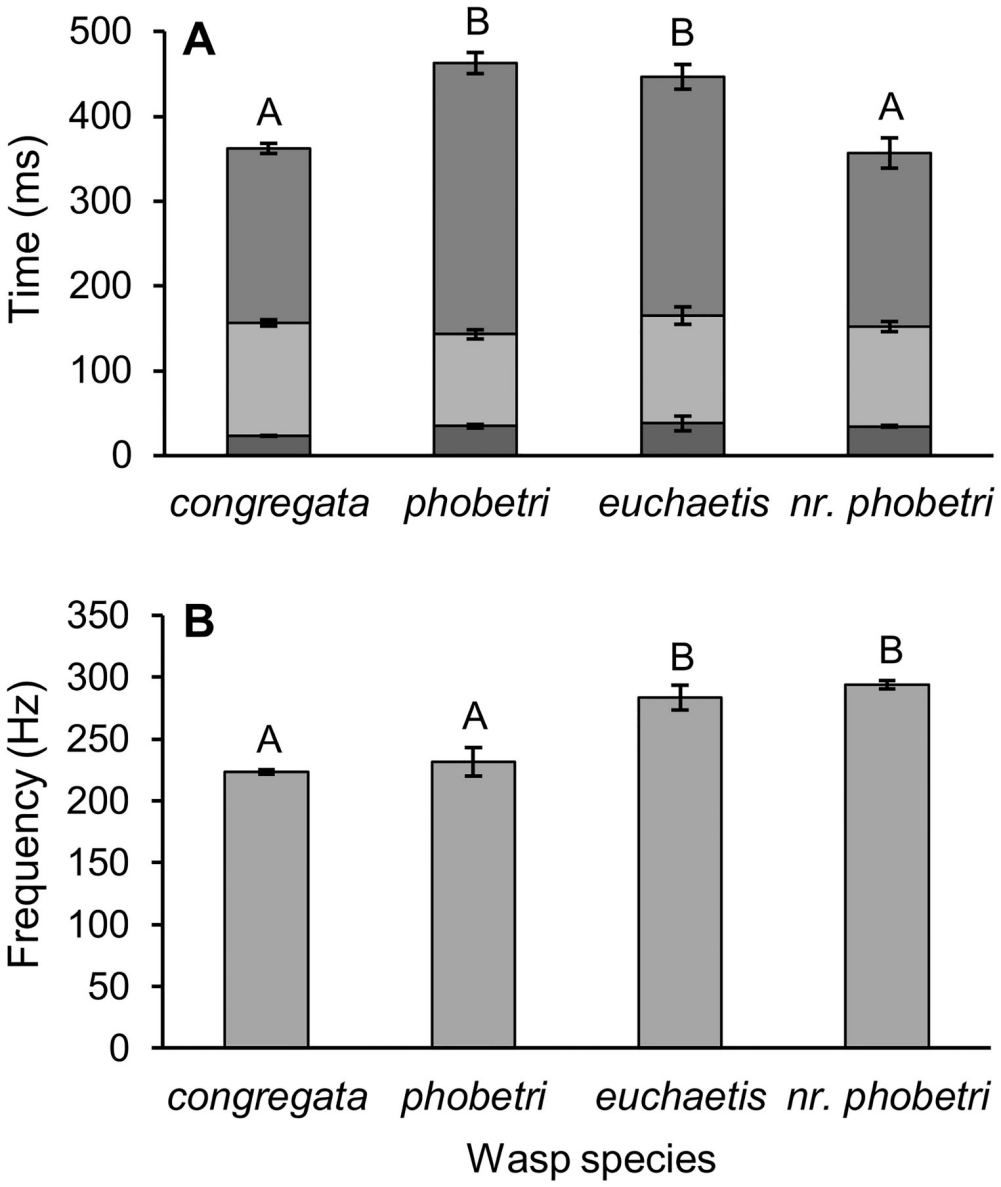
(A) Mean (± SE) duration of interpulse interval divided into pause (bottom), pulse (middle), and terminal buzz (top) elements and (B) fundamental frequency for four *Cotesia* species with similar patterns of song structure in the “*rubecula*” phylogenetic group. Letters indicate significant differences (ANOVA followed by Tukey’s post-hoc test, p < 0.05). Species with similar time elements differ in frequency.

The most common courtship song structure included pause-pulse-buzz elements repeating ca 2–3 times a second. A subset of four species with similar pause-pulse-buzz patterns was used to discern more nuanced differences. The songs differed in either duration of interpulse intervals and component elements (ANOVA: F_3,37_ = 8.03, p = 0.0003; Fig 3A) or fundamental frequency (ANOVA: F_3,37_ = 46.08, p < 0.0001; Fig 3B). In *C*. *congregata*, the courtship songs consisted of an initial buzz followed by repeating pulse-buzz elements with a short (20–26 ms) pause, followed by a pulse (“boing”) that decays into a buzz [20]. Courtship songs of *C*. *phobetri* and *C*. *euchaetis* had similar patterns, although terminal buzzes after pulses were longer than in *C*. *congregata*. *Cotesia* nr. *phobetri* songs were similar to those of *C*. *euchaetis* but with shorter terminal buzzes. *Cotesia marginiventris* songs had pulse-buzzes that varied more in duration, producing a warble sound [21,24]. The song of *C*. *marginiventris* did not contain the discrete high-amplitude pulses present in *C*. *congregata*, *C*. *phobetri*, and *C*. *euchaetis*, but otherwise followed a similar pattern. *Cotesia glomerata* was similar but lacked discrete pauses between pulse-buzz elements and had a sudden, <50 ms, power spike at the start of each pulse that was not observed in other species (Fig 2).

Three species produced courtship songs that consisted of rapid repeating pulses without long terminal buzzes. *Cotesia empretiae* produced a pulse train of ~2 seconds that consisted of short repeating pulses with different durations. *Cotesia diacrisiae* produced rapid short pulses at a rate of four per second. *Cotesia orobenae* also produced rapid repeating pulses that were shorter in duration and had longer pauses compared to *C*. *diacrisiae*. These three species could be readily distinguished by waveform patterns (Fig 2).

Three species produced songs with long pauses between pulses. *Cotesia rubecula* produced songs with pulses similar in duration and pacing to those of *C*. *phobetri* and *C*. *congregata* but lacked a terminal buzz. *Cotesia teleae* produced a pulse-buzz element with pauses that were about five times longer than those of the *C*. *congregata* group. The *C*. *flaviconchae* courtship song was substantially different from other *Cotesia* songs. It was the only species that produced songs with a buzz before the high-amplitude pulse (“pre-pulse buzz”) lasting 486 ± 32 ms at 286 ± 2 Hz. The buzz-pulse repeated every 4–9 seconds, while all other songs repeated in less than a second.

### Analysis of all species

**Fig 4:**
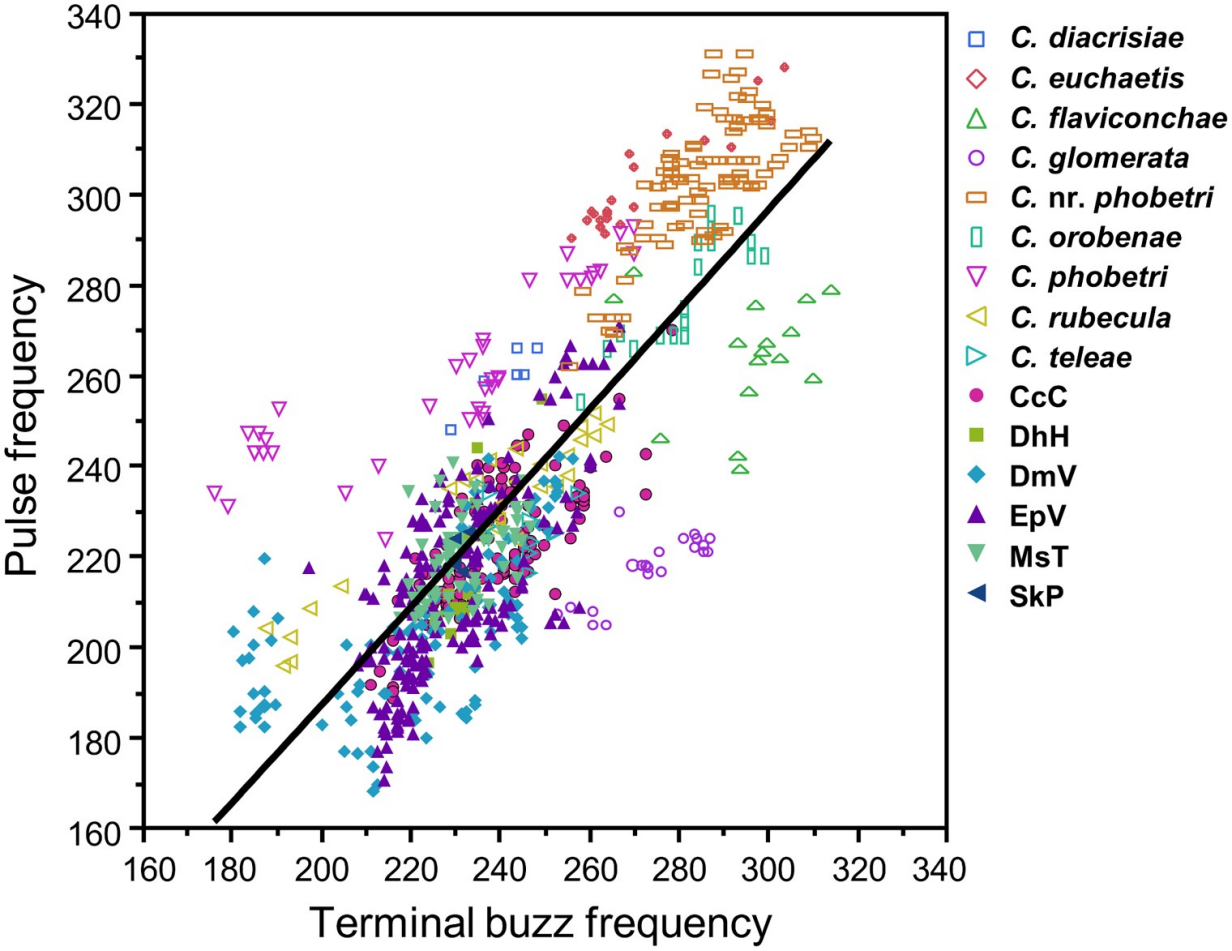
Scatterplot of fundamental frequency (Hz) of adjacent pulse and terminal buzz elements in courtship songs across ten *Cotesia* species and six host-foodplant sources of *C*. *congregata*. Pulses and buzzes were generally correlated in song sections containing both elements (all species, linear regression: *r*^2^ = 0.65, *p* < 0.0001; Pulse = -31.0 + 1.1*TBuzz).

Songs from all 12 *Cotesia* species were grouped using PCA and cluster analysis. Frequency of adjacent pulse and terminal buzz elements could not be accurately calculated in all sections of songs produced by some species due to short durations of one of these elements (e.g., *C*. *empretiae*); however, adjacent pulses and buzzes were correlated in song sections containing sufficiently long durations of both elements (*r*^2^ = 0.65, d.f. = 770, *p* < 0.0001; Pulse = -31.0 + 1.1*TBuzz; Fig 4). Therefore, frequency was consolidated into one term by using the “pulse-buzz unit” frequency in the PCA. The PCA using the seven song elements resulted in four PCs explaining 91.7% of total variance. PC1 was best represented by duration of the pulse-buzz unit, interpulse interval, and pre-pulse buzz, PC2 by frequency, PC3 almost entirely by pause duration, and PC4 by frequency, pause duration, and pulse duration (Table 5). The factor scores of the first four PCs differed significantly among some, but not all, species (ANOVA: F_10,61_ = 83.7, 32.7, 17.1, 27.1 respectively; p < 0.0001; all pairwise comparisons not shown). For example, *C*. *flaviconchae* differs significantly from all other species by PC1 and PC2, whereas species with highly similar song patterns such as *C*. *congregata* and *C*. *phobetri* may only differ by one PC (in this case PC4) but not the others.

**Table 5:** Component matrix (eigenvectors), eigenvalues, and explained variance resulting from principal components analysis of male courtship songs from twelve species of *Cotesia*. Parameters strongly associated with song elements are bold.

	Principal components (PC)			
	1	2	3	4
Pause duration	-0.215	0.100	**0.810**	**0.503**
Pulse-buzz duration	**0.534**	-0.169	0.060	0.086
Pre-pulse buzz duration	0.453	0.413	0.196	-0.221
Interpulse interval	0.467	0.371	0.221	-0.201
Pulse duration	0.361	-0.289	0.006	0.473
Terminal buzz duration	0.334	-0.522	-0.075	0.153
Frequency	0.054	**0.545**	-0.498	**0.635**
Eigenvalue	3.250	1.157	0.989	0.660
Explained variance (%)	46.43	21.68	14.12	9.43
Cumulative variance (%)	46.43	68.11	82.23	91.66

**Fig 5:**
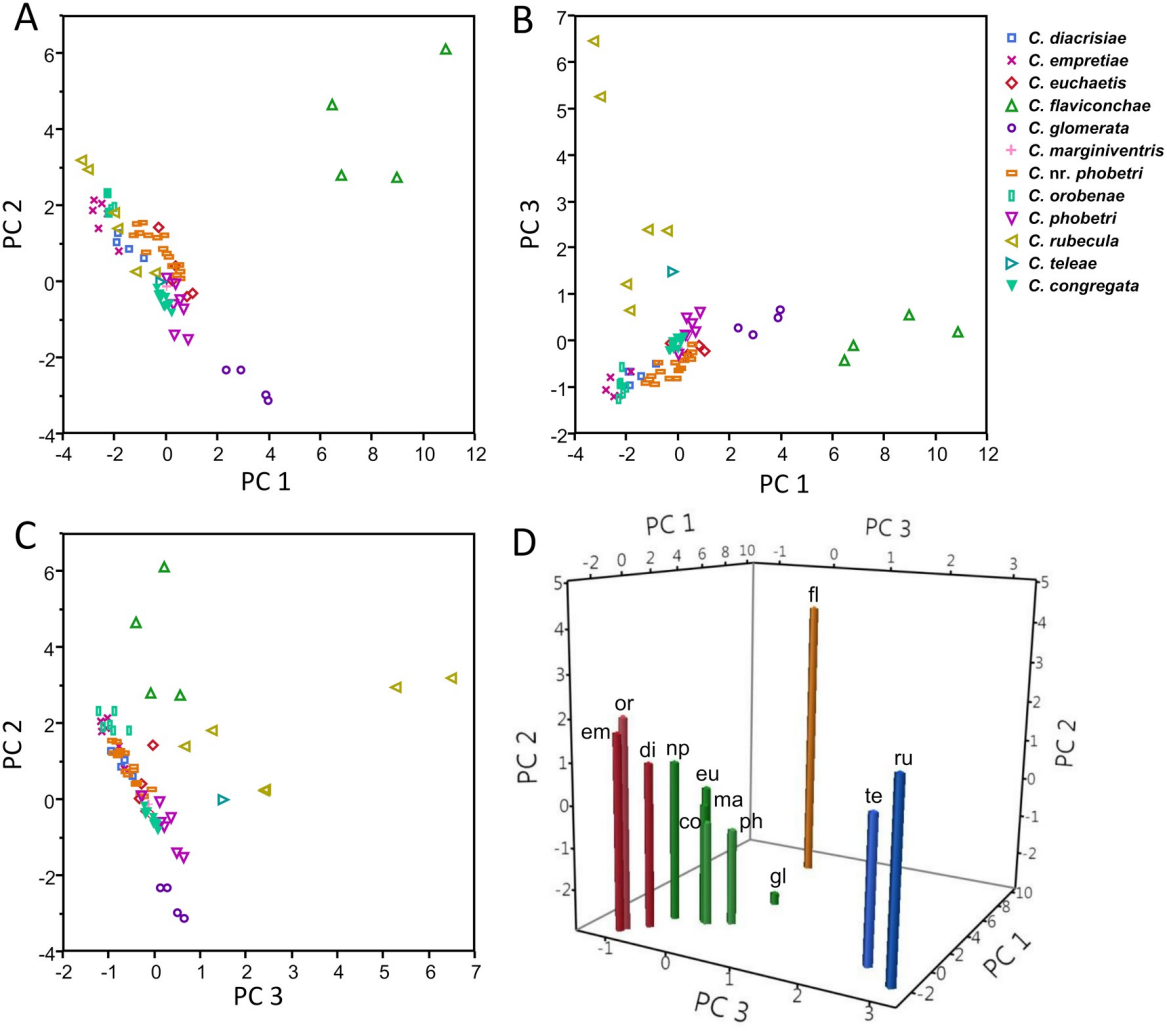
Scatterplots of factors of a principal components analysis of seven *Cotesia* courtship song elements. (A) PC1 vs. PC2, (B) PC1 vs PC3, (C) PC3 vs PC2. Each data point represents a single individual coded by species. (D) 3-dimensional plot of the mean PC of each species color-coded by dominant courtship song pattern (red—rapid pulses; green—pulse-buzz; blue—long pauses; orange—long buzz-pulse). Letter codes indicate species name. *Cotesia congregata* is represented by the MsT host-foodplant complex. Some species are separated from the others by one or more PCs; most species form a close cluster with some overlap.

**Fig 6:**
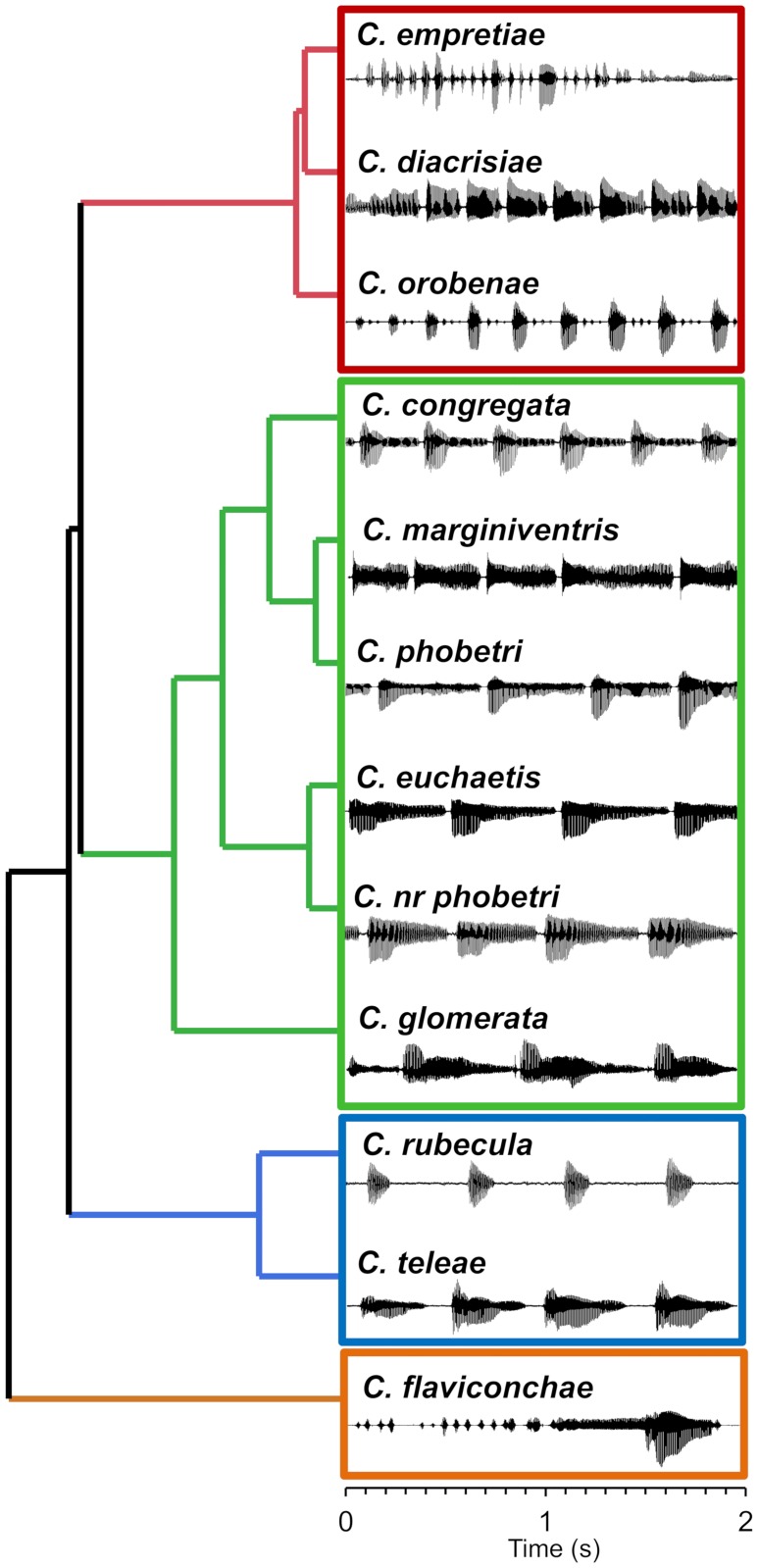
Hierarchical clustering using the first four principal components of seven courtship song elements to group *Cotesia* species. Each species is represented by a 2-second waveform section.

**Fig 7:**
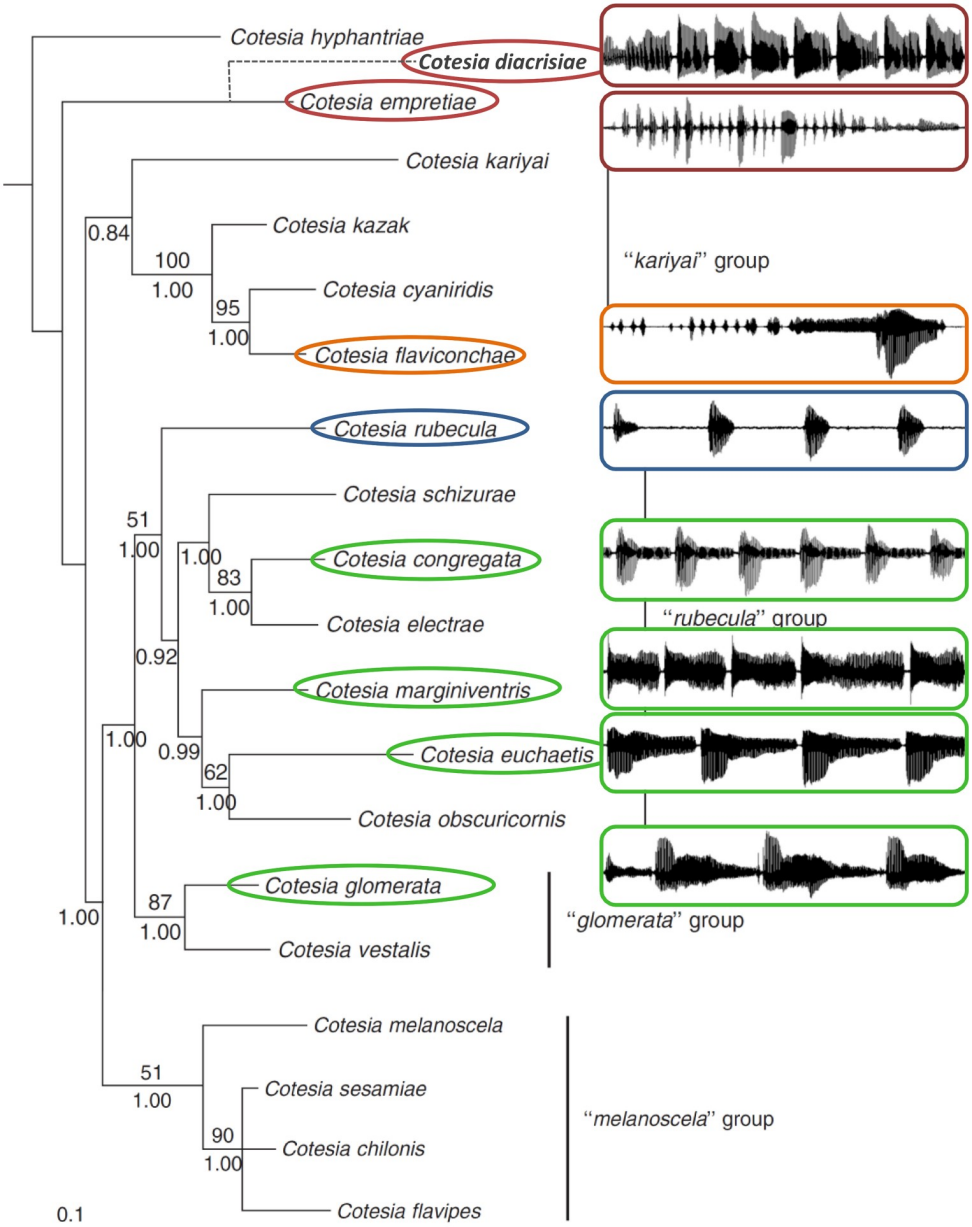
*Cotesia* courtship songs of representative species overlaid on a maximum likelihood tree from the analysis of four genes [44]. Recorded species are in ovals and colors match those in the dendrogram produced by hierarchical cluster analysis using principal components of song elements. *Cotesia diacrisiae* has been added as a sister species of *C*. *empretiae* based on analysis of the NADH1 gene. Nodes have bootstrap values (above, 100 replicates) and Baysian posterior probabilities (below). Non-*Cotesia* outgroups have been removed for clarity (rooted with *Chelonus inanitus*). Courtship song grouping generally follows the genetic phylogeny with the exception of *C*. *rubecula*. The molecular phylogeny was used and modified with permission of J. Whitfield, University of Illinois, USA.

Differences in one or more PCs among species indicated groups with most species forming close clusters with some overlap (Fig 5). Species separated from the main cluster and containing relatively long duration elements had greater variance in PCs (e.g., *C*. *flaviconchae* and *C*. *rubecula*). Hierarchical cluster analysis of species using the first four PC mean factor scores resolved four main groups (Fig 6). Group 1 consists of wasps with short rapid pulses, group 2 with pulses and terminal buzzes, group 3 with long pauses between pulses, and group 4 of only *C*. *flavichonchae* with a thus far unique song pattern. Groupings from the cluster analysis generally reflect genetic groups [44], with the exception of *C*. *rubecula* which lacks the terminal buzz found in all other related species in the “*rubecula*” group (Fig 7).

### Differentiation of *C*. *congregata* host-foodplant complex sources

**Fig 8:**
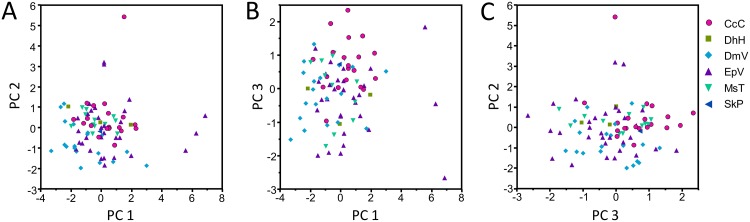
Scatterplots of principal components resulting from principal components analysis of courtship song elements produced by six host-foodplant complex sources of *Cotesia congregata*. (A) PC1 vs. PC2, (B) PC1 vs PC3, (C) PC3 vs PC2. Songs from different host-plant complex sources cannot be reliable distinguished from each other.

**Fig 9:**
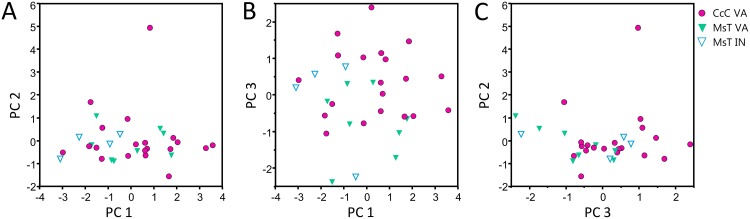
Scatterplots of principal components resulting from principal components analysis of courtship song elements produced by MsT and CcC host-foodplant complex sources of *Cotesia congregata*. (A) PC1 vs. PC2, (B) PC1 vs PC3, (C) PC3 vs PC2. MsT wasps originated from two sources in Virginia and Indiana. Songs from different host-plant complex sources or different locations cannot be reliably distinguished from each other.

Songs from the different *C*. *congregata* host-foodplant complex sources could not be distinguished by courtship songs alone. The PCA using the six song elements present in *C*. *congregata* resulted in three PCs explaining 88.8% of total variance and four PCs explaining 99.9% of total variance. PC1 was most represented by pulse-buzz unit duration, interpulse interval, and terminal buzz duration, PC2 by pause duration and frequency, PC3 by pulse duration, and PC4 by frequency (Table 6). A PCA of the two geographic sources of MsT and the one source of CcC host-foodplant complexes produced a similar component matrix (Table 7). High overlap of every PC prevents discrimination of host-plant complex sources (Fig 8) and geographically separated populations (Fig 9), even if means of some elements and the first threes PCs differ significantly between some groups (ANOVA: p < 0.001).

**Table 6:** Component matrix (eigenvectors), eigenvalues, and explained variance resulting from principal components analysis of male courtship songs from host-foodplant complex sources of *Cotesia congregata*. Parameters strongly associated with song elements are bold.

	Principal components (PC)			
	1	2	3	4
Pause duration	0.146	**0.668**	-0.108	-0.720
Pulse-buzz duration	**0.564**	-0.094	-0.004	0.054
Interpulse interval	**0.567**	-0.048	-0.017	0.016
Pulse duration	0.288	0.048	**0.886**	-0.018
Terminal buzz duration	**0.504**	-0.130	-0.448	0.075
Frequency	0.048	**0.723**	-0.041	**0.688**
Eigenvalue	3.096	1.287	0.943	0.673
Explained variance (%)	51.60	21.45	15.71	11.21
Cumulative variance (%)	51.60	73.05	88.77	99.96

**Table 7:** Component matrix (eigenvectors), eigenvalues, and explained variance resulting from principal components analysis of male courtship songs from MsT and CcC host-foodplant complex sources of *Cotesia congregata*. MsT are divided into wasps originating from Virginia and Indiana. Parameters strongly associated with song elements are bold.

	Principal components (PC)			
	1	2	3	4
Pause duration	0.083	**0.727**	0.036	-0.679
Pulse-buzz duration	**0.584**	-0.008	0.071	0.099
Interpulse interval	**0.586**	0.028	0.055	0.038
Pulse duration	0.233	-0.171	**0.830**	-0.091
Terminal buzz duration	**0.487**	0.103	-0.489	0.175
Frequency	-0.125	**0.657**	0.252	**0.699**
Eigenvalue	2.889	1.224	1.164	0.722
Explained variance (%)	48.14	20.40	19.39	12.03
Cumulative variance (%)	48.14	68.54	87.93	99.96

### Differentiation of *C*. nr. *phobetri* by location

**Fig 10:**
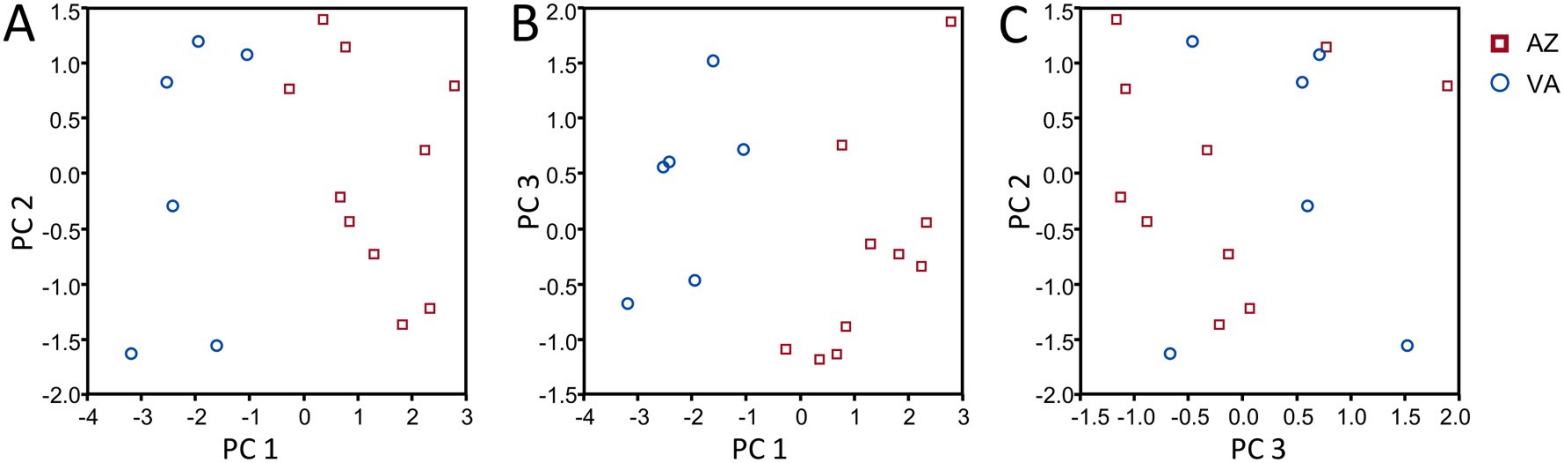
Scatterplots of principal components resulting from principal components analysis of courtship song elements produced by *Cotesia* nr. *phobetri* originating in Arizona and Virginia. (A) PC1 vs. PC2, (B) PC1 vs PC3, (C) PC3 vs PC2. Songs from these two locations can be reliably distinguished from each other based on PC1 (active song element durations), but not PC2 or PC3 (frequency and pause duration). Mean pulse-buzz duration is 0.13 s longer in songs of wasps from Arizona vs. Virginia (t_13_ = -8.6, p < 0.0001).

Songs from the two sources of *C*. nr. *phobetri* could be clearly distinguished by courtship songs. The PCA using the six song elements present in *C*. nr. *phobetri* resulted in three PCs explaining 94.5% of total variance and the fourth PC explaining the remaining variance. PC1 was most represented by duration of pulse and buzz components, PC2 by pause duration and frequency, PC3 by pause duration, and PC4 by pulse duration (Table 8). The *C*. nr. *phobetri* populations from Virginia and Arizona can be reliably distinguished by PC1 (pulse and buzz durations) but not the other PCs (pause duration and frequency) (Fig 10). Linear discriminant analysis sorts wasps by population with 100% accuracy. Mean duration of the pulse and buzz components were longer in songs of wasps originating in Arizona than Virginia (unequal variance t-test, p < 0.001), with mean pulse-buzz unit duration 0.13 s longer (t_13_ = -8.6, p < 0.0001). Overall song pattern and structure remained the same in wasps from both populations and were more similar to each other than to the other wasp species analyzed (Figs 4 and 5).

**Table 8:** Component matrix (eigenvectors), eigenvalues, and explained variance resulting from principal components analysis of male courtship songs from two sources of *Cotesia* nr. *phobetri*: Virginia and Arizona. Parameters strongly associated with song elements are bold.

	Principal components (PC)			
	1	2	3	4
Pause duration	0.117	**0.591**	**0.795**	-0.062
Pulse-buzz duration	**0.520**	-0.032	-0.081	-0.100
Interpulse interval	**0.521**	-0.002	-0.039	-0.112
Pulse duration	**0.447**	-0.183	0.125	**0.809**
Terminal buzz duration	**0.494**	0.041	-0.161	-0.502
Frequency	0.012	**0.784**	-0.565	0.258
Eigenvalue	3.660	1.138	0.873	0.329
Explained variance (%)	61.00	18.96	14.55	5.48
Cumulative variance (%)	61.00	79.96	94.51	99.99

## Discussion

Courtship songs of the twelve species of *Cotesia* presented in this study were variable yet distinguishable. Songs were characterized quantitatively by dividing songs into elements that were shared across most species. Principal components analysis was used to reduce dimensionality of correlated song elements. Species grouped using hierarchical cluster analysis corresponded to groups in the genetic phylogeny [44], with one exception. Some species that have not been sequenced can be provisionally placed into these pre-identified groups based on courtship song characteristics. Likewise, the general pattern of courtship songs may be predicted for species that have been placed within a phylogeny but were not found and recorded in this study. However, the relationships among the groups do not correspond strictly to the genetic phylogeny and the large differences among groups make their placement difficult. Differentiation within *C*. *congregata* by host-foodplant complex was not reliable; in contrast, *C*. nr. *phobetri* could be identified by source. Courtship song analysis has potential use for the systematics of this genus.

Songs were generally unique to each species and could be distinguished by waveform and frequency characteristics. All songs consisted of repeating pulse and/or buzz elements generated from wing fanning, which is common among parasitic wasps. The song pattern was stereotypical for each species (Fig 2 and supplemental audio). Notably, songs of all species had overlapping ranges in the duration and frequency of some elements (Fig 5). Songs of some species with relatively longer elements (e.g., the pause element produced by *C*. *rubecula*) were more variable. The species representing the “*rubecula*” group produced the most similar song patterns (short pause, pulse, terminal buzz) as indicated by tight clustering in the PCA analysis (Fig 5). Songs of some species in this group differed only in the duration or frequency of one or a few elements (Fig 3). Although we attempted to capture the primary acoustic variation among species, some unique characteristics were not integrated into the PCA, e.g., the pre-pulse power spike produced by *C*. *glomerata* and the warble sound produced by *C*. *marginiventris*, which can be used to easily distinguish these species from others with otherwise similar song patterns. We do not rule out that some as yet unrecorded species may produce songs so similar to a sister species as to not be distinguishable, as was the case among the host-foodplant complexes of *C*. *congregata*. Moreover, playback experiments are necessary to determine whether wasps can distinguish between songs of closely related species. Importantly, some clustering of species was expected given the morphological and genetic similarity of many members of *Cotesia*.

Phylogenetics and taxonomy of the Microgastrinae are active areas of study [50]. Most work has been at the subfamily or genus level [51–54], on closely related species clusters or cryptic species [47,55–57], or by determining the evolutionary relationship with symbiotic viruses [58–61]. Considering the diversity of *Cotesia* and the difficulty of producing high-resolution phylogenies, not all of the same genes or all common species have yet been sequenced. Therefore, some species in this study cannot be placed reliably in current phylogenies (e.g., *C*. *phobetri*, *C*. *teleae*). Eight of the recorded species are included in the most recent genetic phylogeny for *Cotesia* reported by Michel-Salzat and Whitfield [44]. This phylogeny contains four identified groups, three of which are represented in our current study. Courtship songs of two species in the fourth group (“*melanoscela*”) have been described in other studies [22,43]. In our study, species phylogenetically grouped together had similar courtship songs with the exception of *C*. *rubecula*. Several species not placed on the genetic phylogeny can be putatively placed within a group based on song characteristics (e.g., *C*. *phobetri*). The most basal genetic group containing *C*. *empretiae* and *C*. *diacrisiae* had songs that consisted of rapid repeated pulses although the placement of these species has low nodal support. Most songs of derived groups consisted of pulses and longer terminal buzzes.

The apparent phylogenetic signal of courtship songs allows for predictions of song structure before recording. For example, most species in the “*rubecula*” group have a pause-pulse-buzz pattern of similar duration (Fig 7). Species such as *C*. *schizurae* and *C*. *electrae* likely have songs similar in pattern to *C*. *congregata* and not more different than the more distantly related *C*. *euchaetis* or *C*. *marginiventris*. In both the genetic phylogeny and acoustic dendrogram, *C*. *glomerata* was placed as a sister group to most of the “*rubecula*” group, with its main distinction being the pre-pulse power spike. In the “*kariyai*” group, *C*. *cyaniridis* is predicted to have a song with a buzz that leads directly into a pulse with a long interpulse duration. The song may differ in details of timing and frequency, but otherwise should sound similar to the closely related *C*. *flaviconchae* (Fig 7). These predictions are supported by the recent recording of two male *C*. *schizurae* (host *Schizura unicornis*; 37.7549, -77.3458) from the same brood. This species, closely related to *C*. *congregata*, was targeted for collection but not initially found. The courtship song of *C*. *schizurae* (S13 Audio) is similar enough in structure and duration of pulses, buzzes, and pauses to that of *C*. *congregata* to be firmly placed as a closely related species. Although courtship songs can be used to construct species groups, predicting evolutionary relationships among these groups is more challenging due to the lack of data for intermediate species. Among other insect groups, phylogenetic signals have been reported for the diverse songs of psyllids, but divergence may occur more rapidly among sympatric species [62]. Predicting the courtship songs of other acoustically active insect groups is challenging due to rapid diversification of song characteristics unrelated to genetic distance (e.g., the *Drosophila willistoni* species complex [3] and *Chrysoperla* lacewings [63]).

*Cotesia rubecula* is the only species that deviates from expectation. It is genetically placed with species with pulse-terminal buzz patterns (e.g., *C*. *congregata*) but lacks a discrete terminal buzz (Fig 2). The most parsimonious explanation is that *C*. *rubecula* secondarily lost the long terminal buzz and replaced it with a long pause. The time between pulses is similar to those within its genetic group. Alternatively, *C*. *rubecula* may not belong in this group, although high nodal support for its inclusion makes this possibility less likely (Fig 7). Notably, *C*. *rubecula* has other characteristics that differentiate it from most species recorded in the “*rubecula*” and “*glomerata*” groups—it is solitary and the largest *Cotesia* species recorded. Determining whether a solitary or gregarious life history or relative size influences courtship song characteristics would require recording a broader range of species, particularly more solitary species (the only other being *C*. *marginiventris* in this study). Moreover, *C*. *rubecula* and *C*. *glomerata* are the only species collected that utilize the same host, *Pieris rapae* on Brassicaceae. Their songs may display greater divergence in part due to character displacement, which has been demonstrated for the songs of few insects [64]; however, extensive surveying suggests competitive exclusion between these two species over most of the range in the United States [65].

*Cotesia teleae* has a song that challenges direct placement into a group. The pattern of the pulse-buzz unit is similar in many ways to those of the “*rubecula*” group; however, it has a short pulse with a high energy terminal buzz that loses amplitude at the end (Fig 2). Most distinctly, there are long pauses between pulses. Possibly, it belongs with the “*rubecula*” group—the cluster analysis supports a relation with *C*. *rubecula*—but its placement remains less certain than for other species without either genetic information or another species with a similar song pattern. The song of *C*. *teleae* is also the only one analyzed using a single male. A single parasitized polyphemus caterpillar (*Antheraea*. *polyphemus*) yielded few adult wasps. The brood began egression in October with most wasp larvae going into diapause, which was not broken in the lab and yielded only a single male concurrently with females. Considering that courtship songs are conserved within species and this male was healthy, the recorded song is presumably a reliable representation of this species. A second male emerged without a living conspecific female present and would not initiate courtship when presented with other species. Attempts to find a second brood over multiple years failed. Since additional samples of *C*. *teleae* are improbable, it was included in this study.

The “*melanoscela*” group, containing *C*. *sesamiae* and *C*. *flavipes*, is the only major group not included in this analysis. These two species, widely used as biocontrol agents of stemborer pests, are not native to North America and could not be acquired for this study. Similar to other *Cotesia spp*., their songs consist of repeating pulse, buzz, and pause elements with a frequency of 222–290 Hz [22,43] (note: our terminology differs from that used in these references). The overall pattern of higher-amplitude pulse decaying into a longer terminal buzz has some structural similarities to songs of *C*. *marginiventris* and others in the “*rubecula*” group; however, the time between high amplitude elements (termed buzz 1 in [22,43]) is nearly a full second in *C*. *flavipes* and *C*. *sesamiae*, which is considerably longer than most of the other *Cotesia* species recorded. The species of the “*melanoscela*” group would form a distinct cluster based on reported song characteristics, and may be placed close to the “*rubecula*” group based on overall pattern.

Courtship song analysis can be used to match unidentified wasps to species, particularly those with similar morphology. Two cases occurred during this study in which parasitoid cocoons were found separated from their host. Unknown *Cotesia* cocoons found on a garden tomato plant were identified as *C*. *orobenae* upon recording. Presumably, the cross-stripped cabbageworm (*Evergestis rimosalis*) hosts had decimated nearby cabbages and had then migrated to the tomato before parasitoid egression. The hosts were absent, leaving only the wasp cocoons remaining on the leaves (*C*. *orobenae* cocoons typically do not remain attached to the host). In the second case, three loose bundles of parasitoid cocoons were found with no host in a mowed horse pasture in Virginia, USA. After recording the adults, the species acoustically matched those grouped with *C*. *congregata* but not any currently recorded species. Subsequently, cocoons of *C*. nr. *phobetri* that originated from Arizona were received. These wasps produced courtship songs that very closely resembled those of the unknown Virginia wasps, and also were similar in morphology, cocoon structure, and habitat. This is the first known record of *C*. nr. *phobetri* outside of Arizona. Considering that this species utilizes a common caterpillar genus, *Grammia*, as hosts in a common habitat type, they may be widespread in the United States.

Courtship song elements may differ even among closely related species or host-associated populations. For example, allopatric populations of *C*. *sesamiae* and *C*. *flavipes* utilizing different hosts had courtship songs that differed in element duration and frequency [43]. Likewise, *C*. *congregata* originating from hosts *M*. *sexta* on tobacco (MsT) and *Ce*. *catalpae* on catalpa (CcC) differed significantly in pulse and pause durations though the differences were not enough to reliably distinguish all individuals [15]. We expanded this earlier finding by using four additional host-foodplant sources of *C*. *congregata* in Virginia and an additional population of MsT wasps from Indiana (Table 1). These six total sphingid host species represent two subfamilies so thus were phylogenetically diverse [66]. Mean song element duration (pulse and pause) and PCs differ among MsT and CcC wasps; however, the degree of range overlap with the additional sources prevents reliable discrimination by source (Figs 8 and 9). The slight differences may indicate recent reproductive isolation that over time may become discrete differences under sexual selection or genetic drift. Breeding crosses using these additional sources of *C*. *congregata* indicate a pattern of asymmetric hybrid female sterility with either MsT or CcC wasps, suggesting only two primary lineages (Bredlau et al., *in submission*).

In contrast, geographically separated populations of *C*. nr. *phobetri* differ in song element durations (Fig 10), even though they are similar enough to be recognized as the same species (S7 and S8 Audio). We cannot determine whether the Virginia and Arizona populations represent sister species, host-associated races, or isolated populations without additional information on reproductive compatibility and range. These populations are separated by 3,150 km and thus may be expected to have some differences in song elements regardless of species status. Another possibility is that laboratory rearing of the Arizona population for three years could have resulted in slight changes in courtship song elements, as reported for other braconid wasps [25]. Collecting wild *C*. nr. *phobetri* at multiple sites would be required to make that assessment. The other geographically separated samples came from *C*. *glomerata* and *C*. *rubecula*; however, not enough individuals were recorded to discern acoustic differences within these species.

Relatively small differences in songs among closely related species indicate a phylogenetic signal that may have useful applications for systematics. Sexual selection likely plays a role in the differentiation of some songs. However, within the majority of the “*rubecula*” group, songs consist of similar pause-pulse-buzz patterns more indicative of a slow build-up of differences rather than active sexual selection. Furthermore, song differences in reproductively isolated incipient species of *C*. *congregata* are slight and cannot be used to reliably identify host-foodplant complex sources. Song differentiation of reproductively isolated species may change over time via genetic drift in the absence of strong sexual selection, producing minor changes in elements among closely related species. In this scenario, songs may seem arbitrarily different among species with relatively small changes, yet still be conserved within species. Courtship shortly after emergence on the natal host-plant may play a role in limiting contact among sympatric, closely related species, thereby reducing selective pressure on courtship song differentiation. Likewise, other factors such as host-plant learning and adaptations to host immune systems may play a greater role in parasitic wasp speciation, leading to differing rates of song differentiation. In contrast, species clusters of *Drosophila* are reported to have large differences in courtship songs, suggesting strong sexual selection leading to differentiation before other traits [2–4,67]. Playback experiments using the *D*. *buzzatii* species cluster demonstrate that females are more likely to accept males with a conspecific song, supporting the role of sexual selection [68]. Likewise, cryptic species complexes of lacewings [8] and sand flies [10] can be reliably distinguished by courtship song patterns.

Courtship songs of *Cotesia* spp. are structurally complex and highly variable compared to those produced by other braconids. For example, courtship songs of other microgastrines vary from the consistent wing fanning sounds that increase in amplitude ca. every 2 seconds produced by *Glyptapantales flavicoxis* [28] (in a genus closely related to *Cotesia* [44]) to the short repeating pulse trains of *Microplitis croceipes* that may merge into a warble [24]. More distantly related braconids, such as those in the Opiinae [24–26], typically produce short repeating pulses similar in structure to the song of *C*. *diacrisiae*. Wasps in the Aphidiinae [27] and Euphorinae (Bredlau, unpublished) produce longer pulses (200–220 ms) with longer pauses between pulses (200–370 ms) that do not trail into terminal buzzes, similar in structure to the song of *C*. *rubecula*. The song of at least one ichneumonid, *Compoletis sonorensis*, also produces a song consisting of pulses (215 ms) and long pauses (215 ms) (Bredlau, unpublished). Among parasitic wasps, constant wing fanning or repeating pulses are the most common patterns. Considering that all *Cotesia* songs consist of pulses in one form or another, the ancestral song most likely also consisted of pulses or consistent wing fanning varying in amplitude that later developed pauses before the high amplitude components. Similarity of songs among species such as *C*. *rubecula* and those in other subfamilies likely evolved independently. For example, convergence of song structure has been reported for allopatric cryptic species of *Chrysoperla* lacewings [69].

The analysis presented in this study has several limitations. Even the relatively simple songs of parasitic wasps contain multiple acoustic elements and frequencies, often with different degrees of variance depending on song structure. Principal component analysis is useful for reducing the dimensionality of complex datasets to uncorrelated variables, and in identifying elements that contain the greatest variance. Moreover, PCA is a common method of data exploration widely understood by biologists and has been used in the comparison of songs in diverse taxa including birds (e.g. [70,71]) and insects [4,8,10,63,72]. We used PCA as a means to reduce the acoustic data for comparison and included the number of PCs that adequately explained aspects of the courtship songs in the cluster analysis. However, limitations such as uneven scaling of long vs short elements and time vs frequency were not accounted for because their biological significance is unknown. No one element could adequately capture the differences among songs; furthermore, some elements calculated were intentionally redundant. Alternative methods to a cluster analysis that consider the probability of a given acoustic tree among all possible trees should be considered with additional data. Additionally, the sequencing of all sampled wasps with additional genes or recordings of those already sequenced will permit a more thorough examination of song trait evolution.

This comprehensive study of courtship song diversity within a genus of parasitic wasps, *Cotesia*, implicates a wide diversity of song patterns that can be divided into groups based on duration and frequency of song elements. The basal song most likely consisted of regular pulses generated by high-amplitude wing strokes, as seen in other members of subfamily Microgastrinae [24]. This song diverged into the several distinct patterns among the major groups of *Cotesia*. Many wasps not yet recorded can likely be placed into these groups based on a combination of song structure and morphology. The unique structure of songs for each species can potentially be used for species recognition and as a reproductive barrier between cryptic species; however, the influence of sexual selection is uncertain. Despite measurable differences among species, the songs among *C*. *congregata* host-foodplant complexes cannot be reliable distinguished, suggesting that song differentiation does not proceed without other reproductive barriers. In total, fifteen *Cotesia* species have been recorded out of the estimated 1,000 species globally [50]. Considering the size of this genus, other entirely new song patterns may yet be discovered. When combined with additional genetic data, courtship song analysis should prove useful in determining the systematics and evolutionary history of groups of parasitic wasps, particularly in this highly diverse and agriculturally important taxon.

## Supporting information

S1 Audio Audio recording of a courtship song segment (10 s) of a male *Cotesia congregata* (host *Manduca sexta* on tobacco from Indiana, USA population). (WAV)

S2 Audio Audio recording of a courtship song segment (10 s) of a male *Cotesia diacrisiae*. (WAV)

S3 Audio Audio recording of a courtship song segment (10 s) of a male *Cotesia empretiae*. (WAV)

S4 Audio Audio recording of a courtship song segment (10 s) of a male *Cotesia euchaetis*. (WAV)

S5 Audio Audio recording of a courtship song segment (15 s) of a male *Cotesia flaviconchae*. (WAV)

S6 Audio Audio recording of a courtship song segment (10 s) of a male *Cotesia glomerata*. (WAV)

S7 Audio Audio recording of a courtship song segment (10 s) of a male *Cotesia* nr. *phobetri* (Arizona, USA population). (WAV)

S8 Audio Audio recording of a courtship song segment (10 s) of a male *Cotesia* nr. *phobetri* (Virginia, USA population). (WAV)

S9 Audio Audio recording of a courtship song segment (10 s) of a male *Cotesia orobenae*. (WAV)

S10 Audio Audio recording of a courtship song segment (10 s) of a male *Cotesia phobetri*. (WAV)

S11 Audio Audio recording of a courtship song segment (10 s) of a male *Cotesia rubecula*. (WAV)

S12 Audio Audio recording of a courtship song segment (10 s) of a male *Cotesia teleae*. (WAV)

S13 Audio Audio recording of a courtship song segment (10 s) of a male *Cotesia schizurae*. This species was not included in the analysis.
(WAV)

S1 Appendix Mean song element duration and frequency by wasp for different *Cotesia* species. (XLSX)
